# Real-world survival patterns and multimodal therapy utilization in small cell lung cancer: a retrospective cohort study in a Chinese countryside hospital

**DOI:** 10.3389/fonc.2025.1636533

**Published:** 2025-10-23

**Authors:** Taosheng Huang, Linjie Ma, Mingfang Wang, Xumei Pang, Jiying Xu, Xi Chen, Yutao Xia, Min Yan, Wenxiang Zhao, Congcong Cheng, Runqing Wang, Kai Sun, Peng Wang

**Affiliations:** Department of Oncology, Yidu Central Hospital of Weifang, Weifang, China

**Keywords:** SCLC - small cell lung cancer, radiotherapy, chemotherapy, CCRT, prophylactic cranial irradiation (PCI)

## Abstract

**Introduction:**

Small cell lung cancer (SCLC) accounts for 13–15% of all lung malignancies and remains a highly aggressive disease with limited therapeutic progress, particularly in rural settings. Despite advances such as immune checkpoint inhibitors and multimodal therapy, real-world evidence on treatment utilization and survival outcomes in developing regions is scarce. This retrospective cohort study aimed to evaluate survival patterns and multimodal therapy use in SCLC patients from a Chinese countryside hospital.

**Methods:**

A total of 132 patients diagnosed with SCLC at Weifang Yidu Central Hospital between 2014 and 2023 were retrospectively analyzed. Patients were classified as limited-stage (LS) or extensive-stage (ES) using the Veterans Administration Lung Study Group (VALG) system. Clinical data, including demographics, treatment regimens, and outcomes, were collected. Overall survival (OS) and progression-free survival (PFS) were calculated using the Kaplan-Meier method, and subgroup analyses assessed the effects of platinum sensitivity, prophylactic cranial irradiation (PCI), concurrent chemoradiotherapy (CCRT), and palliative radiotherapy.

**Results:**

Of 132 patients (64 LS-SCLC, 68 ES-SCLC), most received first-line platinum–etoposide regimens, with diminishing treatment continuity in later lines due to cumulative toxicities. Median OS was significantly longer in LS-SCLC than ES-SCLC (50.2 vs. 16.8 months, p<0.05). PCI reduced brain metastases (0% vs. 27%) and improved OS (50.2 vs. 36.4 months; HR=0.47), though not statistically significant. CCRT achieved longer OS than sequential chemoradiotherapy (54.9 vs. 50.2 months; HR=0.54). In second-line therapy, platinum-sensitive patients benefited from platinum rechallenge (median OS 17.7 vs. 12.5 months, p<0.05), whereas platinum-resistant patients showed no improvement. Palliative radiotherapy in ES-SCLC prolonged PFS (16.1 vs. 7.8 months) and OS (30.2 vs. 16.1 months) with near-significant trends (HR≈0.5).

**Discussion:**

This real-world study confirms that concurrent chemoradiotherapy (CCRT) and prophylactic cranial irradiation (PCI) confer survival advantages in LS-SCLC, while palliative radiotherapy yields potential benefits in ES-SCLC. Platinum sensitivity remains a crucial predictor of second-line treatment efficacy, supporting reintroduction of platinum in sensitive relapses per guideline recommendations. Conversely, irinotecan–lobaplatin combinations provided limited benefit. Findings emphasize the need for personalized treatment sequencing and improved access to standardized multimodal care in rural healthcare settings.

## 1 Introduction

Small cell lung cancer (SCLC), representing 13-15% of global pulmonary malignancies,. This aggressive neuroendocrine carcinoma demonstrates remarkable etiological dependence on tobacco exposure in 94% of cases (1, 2). Unlike in high-income countries, there is a rising environmental carcinogen exposure that contributes to increasing disease burden in developing regions (3).SCLC is usually classified into two stage, extensive-stage SCLC (ES-SCLC)and limited-stage SCLC (LS-SCLC), according to staging system of the Veterans Administration Lung Study Group (VALG) instead of Tumor, Node, Metastasis (TNM) staging system.Based on VALG stage system, appropriate treatment model could be recommend to patients (4).

Platinum-etoposide chemotherapy combined with thoracic radiotherapy remains the cornerstone for LS-SCLC, achieving 20-25% of 5-year survival rates. The immunotherapy revolution has reshaped ES-SCLC management, where the addition of PD-L1/PD-1 inhibitors (atezolizumab/durvalumab) to first-line chemotherapy demonstrated unprecedented survival benefits in pivotal phase III trials (5, 6). Nevertheless, disease progression in over 80% of responders occurs within 12 months due to the limited efficacy and rapidly acquired chemoresistance (7).The survival benefits derived from subsequent-line treatments remain constrained.leaves less than 7% of patients with 5-year survival rates (8).

Clinical implementation of therapies faces multifaceted barriers in countryside area of China, despite guideline recommendations from Chinese Society of Clinical Oncology(CSCO) (9) and NCCN (10). We have found that factors such as the accuracy of disease staging, standardization of treatment protocols, consistency in efficacy evaluation, effective management of adverse reactions, and patient compliance significantly influence treatment outcomes.Therefore, it is necessary to obtain a comprehensive analysis of the real-world evidence for its clinical multimodal therapy utilization in developing country,especially rural area.

This multicenter retrospective cohort study (N = 132; 2014-2023),systematically evaluates survival outcomes, treatment attrition patterns, and multimodal therapeutic efficacy in real-world SCLC management. By elucidating the determinants of therapeutic failure across disease stages and healthcare settings, our findings aim to address critical knowledge gaps in treatment sequencing strategies while informing health policy interventions to mitigate global disparities in SCLC care.

## 2 Method

### 2.1 Study design and patient cohort

This retrospective cohort study analyzed 132 patients diagnosed with SCLC who received treatment at a single institution between 2014 and 2023. Patients were stratified into LS and ES disease based on the VALG staging system.LS-SCLC is limited to one hemithorax by imaging and/or by whether the disease is treatable with a tolerable radiation field. ES-SCLC is any disease beyond the boundaries of the limited stage, which corresponds to the stage IV disease of the TNM staging system (4). Inclusion criteria necessitated a systemic baseline examination, including contrast CT scans of the chest and abdomen, as well as a sonographic examination of the neck for the detection of metastatic lymph nodes. It was imperative that patients had histologically confirmed small - cell lung cancer (SCLC) and had received at least one line of systemic therapy. Patients with incomplete medical records or concurrent malignancies were excluded.

### 2.2 Data collection and variables

Clinical data, including demographics, staging, treatment regimens (chemotherapy, radiotherapy), toxicities, and survival outcomes, were extracted from electronic medical records from Weifang Yidu Central Hospital. Performance status (PS) was assessed using the Eastern Cooperative Oncology Group (ECOG) scale. Treatment lines were categorized as first-line to fourth-line based on sequential administration after prior therapy failure. Radiotherapy modalities included Prophylactic cranial irradiation (PCI), thoracic intensity-modulated radiotherapy, and palliative radiation for metastases.All treatment related side effects were described as Common Terminology Criteria for Adverse Events (CTCAE) Version 5.0.

### 2.3 Outcome measures

Primary endpoints were overall survival (OS), defined as time from diagnosis to death from any cause, and progression-free survival (PFS), defined as time from treatment initiation to disease progression or death.

### 2.4 Statistical analysis

Survival curves were generated using the Kaplan-Meier method and compared via the log-rank test. Cox proportional hazards regression models were used to estimate HRs with 95% confidence interval (CI) for variables including disease stage, treatment modality, and radiotherapy use. Subgroup analyses were compared between platinum-sensitive and resistant cohorts, CCRT and Short-Course Radiotherapy (SCRT). All statistical analyses were conducted using R software, with a p-value threshold of < 0.05 indicating statistical significance.

### 2.5 Ethical considerations

The study protocol was approved by the institutional review board in Weifang Yidu Central Hospital, which waived informed consent due to the retrospective design.

## 3 Result

### 3.1 The character of enrolled and the treatment SCLC patients

A total of 161 patients diagnosed with small - cell lung cancer (SCLC) were recruited. Among the non - enrolled patients, 11 were diagnosed with complex pulmonary malignancies, including lung adenocarcinoma and small - cell lung cancer. 9 patients chose to receive palliative treatments such as traditional Chinese medicine. 7 patients lacked comprehensive baseline examinations. 2 patients had no records of treatment regimens. Ultimately, 132 SCLC patients were included in our subsequent analysis(**Supplementary Figure 1**).Those 132 patients were most aged people (age > 60), male is the dominated population than female.About performance score, most of the patients were great (PS ≤1).Only 6 patients were seem as early stage SCLC.The detail of baseline is showed in supplement document 1.

In our retrospective analysis of 132 SCLC patients treated at Weifang Central Hospital between 2014 and 2024, 68 patients were classified as having extensive - stage disease and 64 patients were classified as having limited - stage disease. First - line regimens consisted of etoposide/carboplatin (EC, n = 45), etoposide/cisplatin (EP, n = 74), etoposide/lobaplatin (EL, n = 6), irinotecan/cisplatin (IP, n = 6), and etoposide monotherapy (n = 1). Subsequent treatment dropout analysis revealed that 96 patients received second - line therapy, including irinotecan (n = 66), etoposide (n = 25), anlotinib (n = 2), and paclitaxel (n = 3). In the group that received third - line therapy, the total number of patients decreased to 51, with patients receiving anlotinib (n = 15), irinotecan (n = 16), anlotinib - taxane combinations (n = 11), etoposide (n = 6), and taxane (n = 3). In the fourth - line therapy, 25 patients received anlotinib (n = 8), taxane (n = 8), etoposide (n = 3), irinotecan (n = 3), and mitomycin (n = 3), respectively. Notably, cumulative treatment toxicities from multimodal therapies gradually impaired patients’ performance status. Persistent appetite loss led to severe nutritional depletion, which significantly restricted patients’ tolerance for subsequent therapies, resulting in reduced enrollment in advanced treatment lines after the failure of prior regimens.

### 3.2 Comparison of survival outcomes and treatment efficacy in limited-stage and extensive-stage small cell lung cancer

#### 3.2.1 LS-SCLC demonstrates superior survival outcomes over extensive-stage

**Figure 1:**
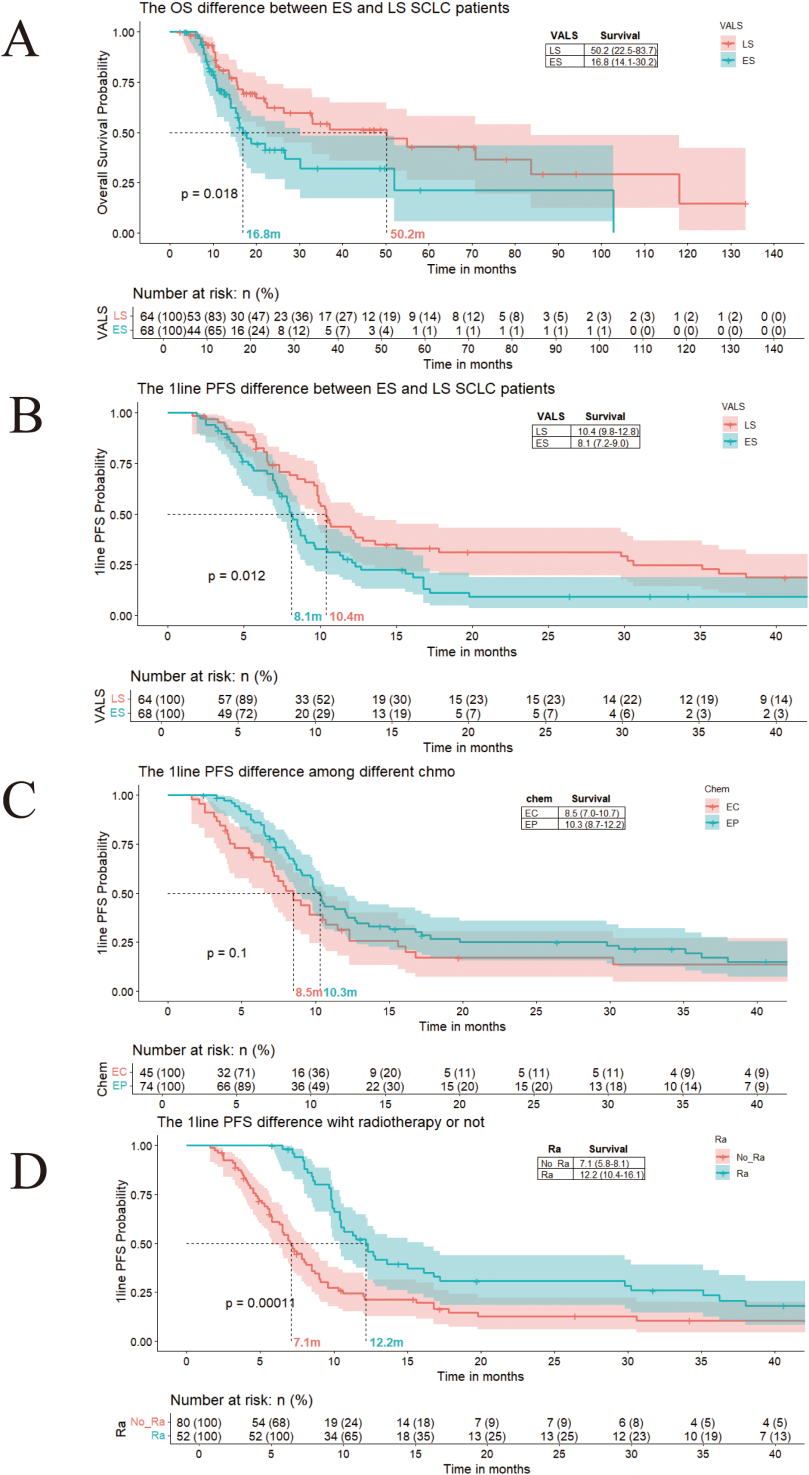
**(A)** The overall survival difference between ES and LS SCLC patients. **(B)** The first-line PFS difference between ES and LS SCLC patients. **(C)** The first-line PFS difference between SCLC patients with EC and EP. **(D)** The first-line PFS difference between SCLC patients with or without radiotherapy.

Patients with limited-stage disease demonstrated significantly longer median OS (50.2 months, 95% CI: 22.5–83.7) compared to those with extensive-stage disease (16.8 months, 95% CI: 14.1–30.2) (**Figure 1A**). Similarly, first-line median PFS was notably longer in limited-stage patients (10.4 months, 95% CI: 9.8–12.8) versus extensive-stage patients (8.1 months, 95% CI: 7.2–9.0) (**Figure 1B**). These findings underscore the importance of early detection and treatment initiation in SCLC.

#### 3.2.2 First-line treatment outcomes

Patients predominantly received EC or EP regimens. Median PFS for EC-treated patients was 8.5 months (95% CI: 7–10.7), while EP-treated patients achieved a median PFS of 10.3 months (95% CI: 8.7–12.2), with no statistically significant difference between the two regimens (p>0.05) (**Figure 1C**). Patients who received radiotherapy (including PCI, cranial or thoracic intensity-modulated radiotherapy, or radiotherapy for isolated metastases) exhibited a prolonged median PFS of 12.2 months compared to 7.1 months in non-radiotherapy patients (**Figure 1D**). This results suggest potential survival benefits from combined modality therapy even though there is a treatment choose bias such as LS-SCLC always receive radiotherapy.

#### 3.2.3 Second-line treatment outcomes

**Figure 2:**
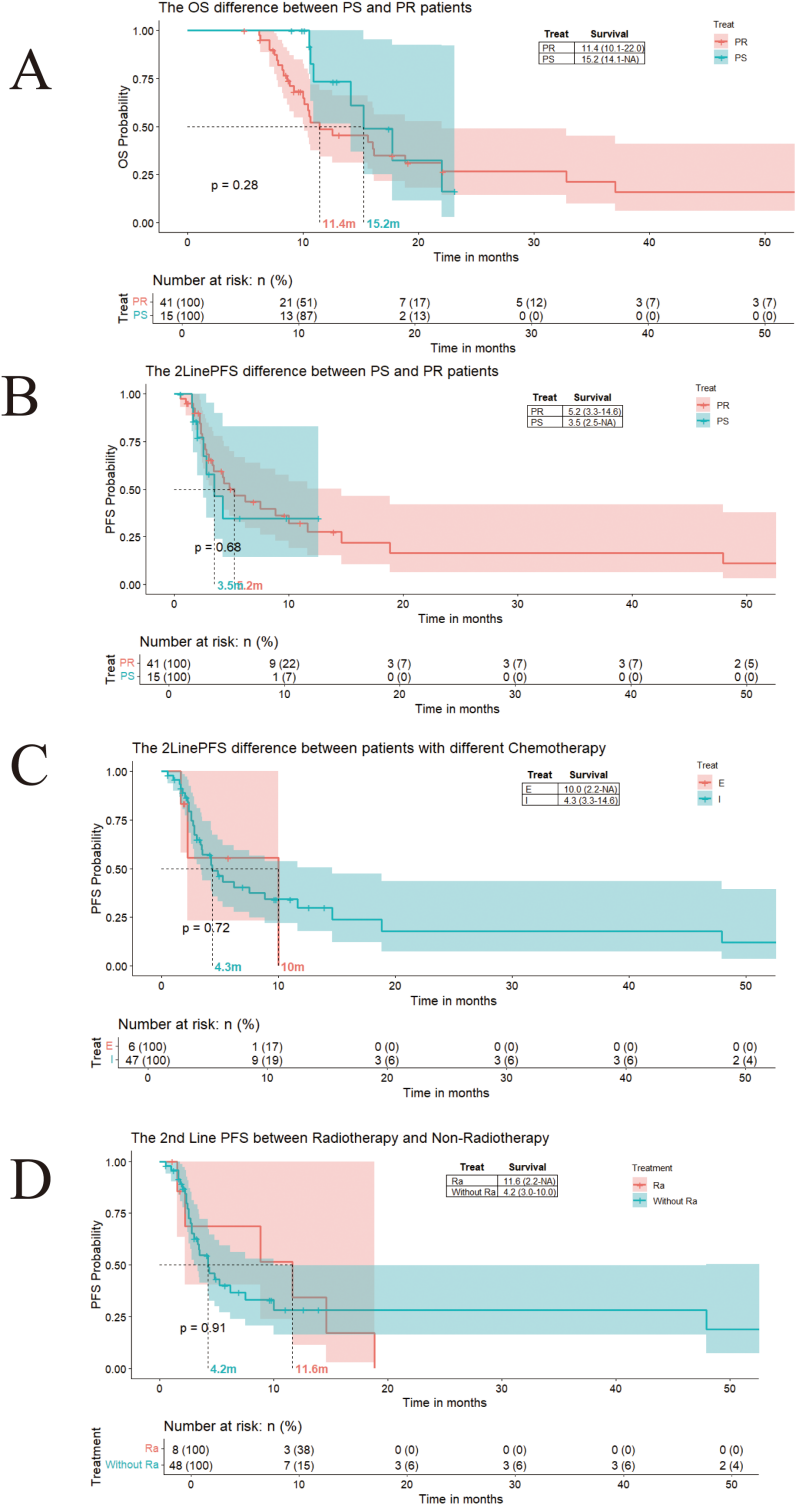
**(A)** The OS difference between platinum-resistant patients and platinum-sensitive patients. **(B)** The second-line PFS difference between resistant patients and platinum-sensitive patients. **(C)** The second-line PFS difference between SCLC patients with Etopostide therapy and Irinotecan therapy. **(D)** The second-line PFS difference between SCLC patients with or without radiotherapy.

After first line treatment, there are 96 patients who accept second line treatment in our cohort. As the Log-Rank curve showing, the media PFS for the second line treatment of those patients is 4.3 months. Based on European Society for Medical Oncology(ESMO) guidelines, patients were stratified into platinum-sensitive (treatment-free interval ≥90 days while <180 days, PS) and platinum-resistant (treatment-free interval <90 days, PR) cohorts (11). Platinum-sensitive patients exhibited superior median OS (15.2months) versus platinum-resistant patients (11.4 months) (**Figure 2A**). However, no significant difference was observed in second-line PFS between the PS groups (3.4 months, 95% CI: 2.5–5.2) and the PR groups (4.1 months, 95% CI: 2.8–5.1) (**Figure 2B**). Second-line regimens primarily included etoposide (10 months), or irinotecan (4.3 months) chemotherapy, showing no statistically significant difference in median PFS (**Figure 2C**). Patients receiving radiotherapy during second-line therapy showed a numerically higher median PFS (11.6 months vs. 4.2 months), though statistical significance was not achieved (**Figure 2D**).

**Figure 3:**
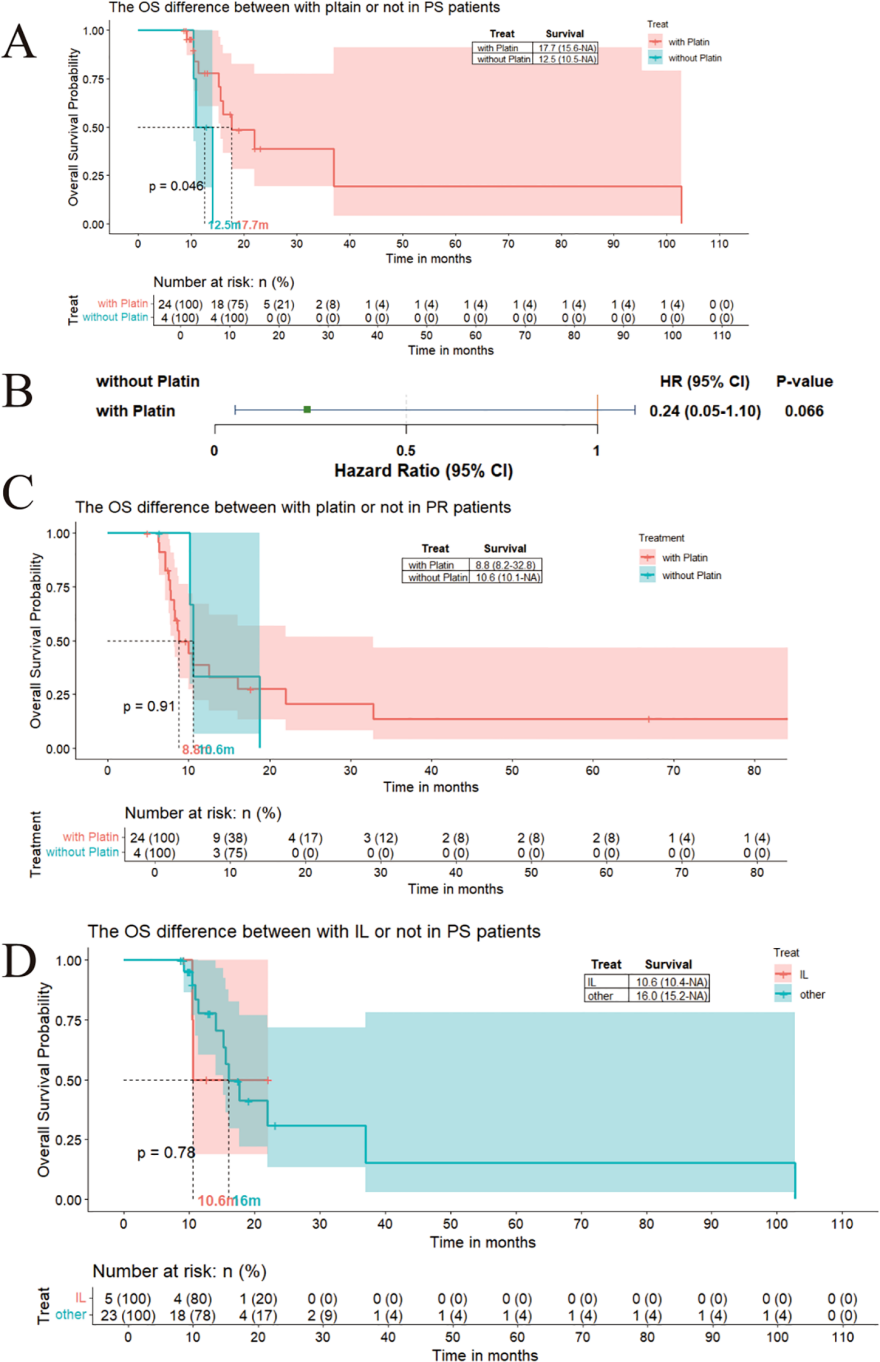
**(A)** The mOS of patients receive platinum as the second line treatment is 17.7 months verse 12.5monts without platinum in PS patients; **(B)** The Cox result to show the HR for with platinum compering to without platinum in PS patients; **(C)** The mOS of patients receive platinum as the second line treatment is 10.6 months verse 8.8months without platinum in PR patients; **(D)** The mOS for IL in second line treatment is 10.6 months verse 16 months in PS patients.

**Figure 4:**
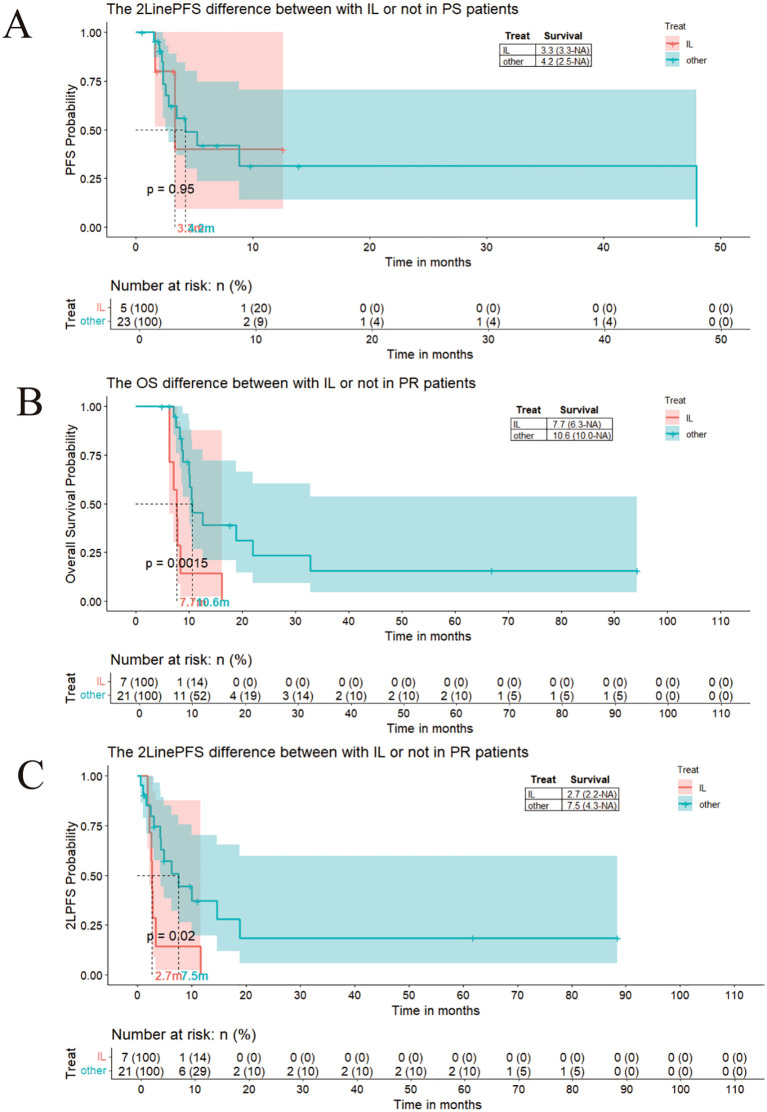
**(A)** The mPFS for IL in second line treatment is 3.3 months verse 4.2 months in PS patients; **(B)** The mOS for IL in second line treatment is 7.7months verse 10.6 months in PR patients; **(C)** The mPFS for IL in second line treatment is 2.7 months verse 7.5 months in PR patients.

As motioned previously, the patients needing the second-line patients could stratified as platinum-sensitive group and platinum-resistance group.We compared the outcome based on different chemotherapy regimens in PR and PS groups. In the platinum-sensitive (PS) group, patients receiving a platinum-based regimen as second-line treatment demonstrated a numerically superior, albeit statistically non-significant, trend towards improved overall survival (OS) (17.7 months vs. 12.5 months, p < 0.05; **Figure 3A**). According to Cox proportional hazards analysis, treatment with platinum was associated with a 76% reduction in the risk of death compared to non-platinum regimens (**Figure 3B**). In contrast, within the platinum-resistant (PR) group, the addition of platinum did not significantly alter clinical outcomes (8.8 months with platinum vs. 10.6 months without platinum, P > 0.05; **Figure 3C**). The irinotecan-lobaplatin (IL) combination was identified as a potential platinum-alternative regimen for SCLC patients who had experienced failure of first-line therapy.Prophylactic Cranial Irradiation (PCI) demonstrates a numerically superior, albeit statistically non-significant, trend toward improved survival in patients with small cell lung cancer.Accompanied by treatment development, now there are others regiment for progressive SCLC patients, including IP(Irinotecan-Cisplatin),irinotecan or etoposide monotherapy,paclitaxel.IL regimen could not extent the OS and PFS both in PS and PR groups, even with a impede survival trend (**Figure 3D**, **Figures 4A–C**).

#### 3.2.4 Third-line treatment outcomes

**Figure 5:**
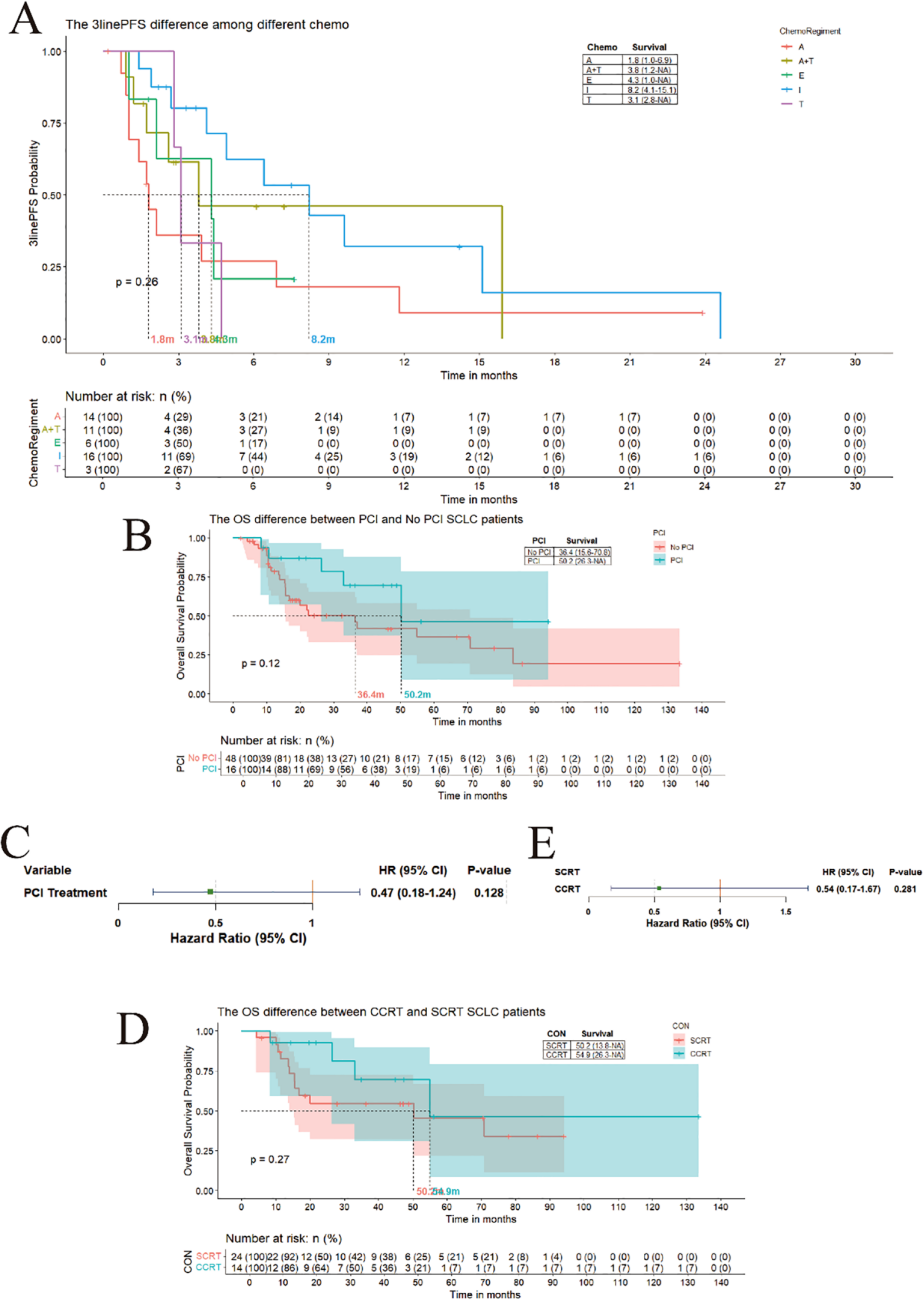
**(A)** The difference in third-line PFS among different regimens. **(B)** The difference of OS between LS-SCLC patients with PCI and not; **(C)** The hazard of PCI treatment compare to no PCI; **(D)** The difference of OS between LS-SCLC patients with CCRT and SCRT;E:The hazard of CCRT treatment compare to SCRT.

Third-line regimens included monotherapies anlotinib, nab-paclitaxel, irinotecan, etoposide, or combination therapy of anlotinib and nab-paclitaxel. Median PFS in all groups is less than 9 months, including 1.8 months for anlotinib, 4.3 months for etoposide, 8.2 months for irinotecan, 3.1 months for nab-paclitaxel, and 3.8 months for the combination therapy of anlotinib and nab-paclitaxel. No significant differences were observed among the five regimens (**Figure 5A**).

### 3.3 PCI demonstrates clinically meaningful survival trends in small cell lung cancer

In patients with SCLC, PCI following chemotherapy has been shown to reduce the incidence of brain metastases in both LS and ES disease. In our hospital, the protocol for PCI involves a radiotherapy dose of 2.5 Gy per fraction, with a total cumulative dose of 25 Gy. Patients are scheduled to complete the PCI course within 12 days. However, whether this reduction translates into survival benefits remains controversial. We retrospectively analyzed survival outcomes in patients who received PCI versus those who did not. In our cohort, there were 64 patients diagnosed with limited - stage small - cell lung cancer (SCLC). As suggested by the guidelines, these patients could potentially benefit from radiotherapy, such as PCI. Among these 64 patients, 16 received PCI treatment, while 48 did not. The majority of these patients experienced treatment failure in the first - line therapy. No patients who received PCI developed brain metastasis. In contrast, among the patients who did not receive PCI, 13 developed brain metastasis. Based on this data, it can be inferred that the occurrence of brain metastasis is associated with non - receipt of PCI (**Supplementary Figure 2**). The median OS was 50.2 months in the PCI group compared to 36.4 months in the non-PCI group (**Figure 5B**). Although this difference appeared clinically meaningful, it did not reach statistical significance. Cox proportional hazards regression analysis demonstrated an HR of 0.47 (95% CI: 0.14–1.24) for PCI versus non-PCI (**Figure 5C**). Our findings suggest that PCI may confer a survival advantage, with reduced brain metastasis rates potentially translating into OS benefits. Patients receiving PCI exhibited a trend toward improved survival outcomes. Regarding the failure of first-line treatment for LS - SCLC, patients who underwent PCI typically experienced disease progression due to metastasis in other organs, such as liver metastasis. A significant number of patients who did not receive PCI ultimately developed brain metastasis. The stage and treatment failure details of LS - SCLC patients were documented in **Supplementary Document 2**.

### 3.4 Concurrent chemoradiotherapy shows clinically meaningful survival trend over sequential chemoradiotherapy in LS-SCLC

For LS-SCLC, CCRT is internationally recommended as the standard of care due to enhanced local tumor control and systemic micrometastasis suppression through synergistic effects. In our hospital, the concurrent chemoradiotherapy (CCRT) protocol entails a radiotherapy dosage of 2 Gy per fraction, with a total cumulative dosage of 30 Gy. Patients are arranged to finish the CCRT course within approximately one month. However, CCRT is associated with higher rates of adverse events, including radiation esophagitis, myelosuppression, and pneumonitis. SCRT, administered in distinct phases, offers better tolerability for patients with poor performance status (PS) or comorbidities. We compared survival outcomes between CCRT and SCRT. The median OS was 54.9 months for CCRT versus 50.2 months for SCRT. Cox regression analysis yielded an HR of 0.54 (95% CI: 0.17–1.67) for CCRT relative to SCRT (**Figures 5D, E**). Although the statistical significance is lacking, our data suggest a clinically significant trend that favors concurrent chemoradiotherapy (CCRT) for the improvement of overall survival (OS). These findings are consistent with the recommendations of guidelines and support CCRT as the preferred approach for eligible limited-stage small cell lung cancer (LS - SCLC) patients.

### 3.5 Symptom-directed radiotherapy associates with prolonged survival trends in ES-SCLC

**Figure 6:**
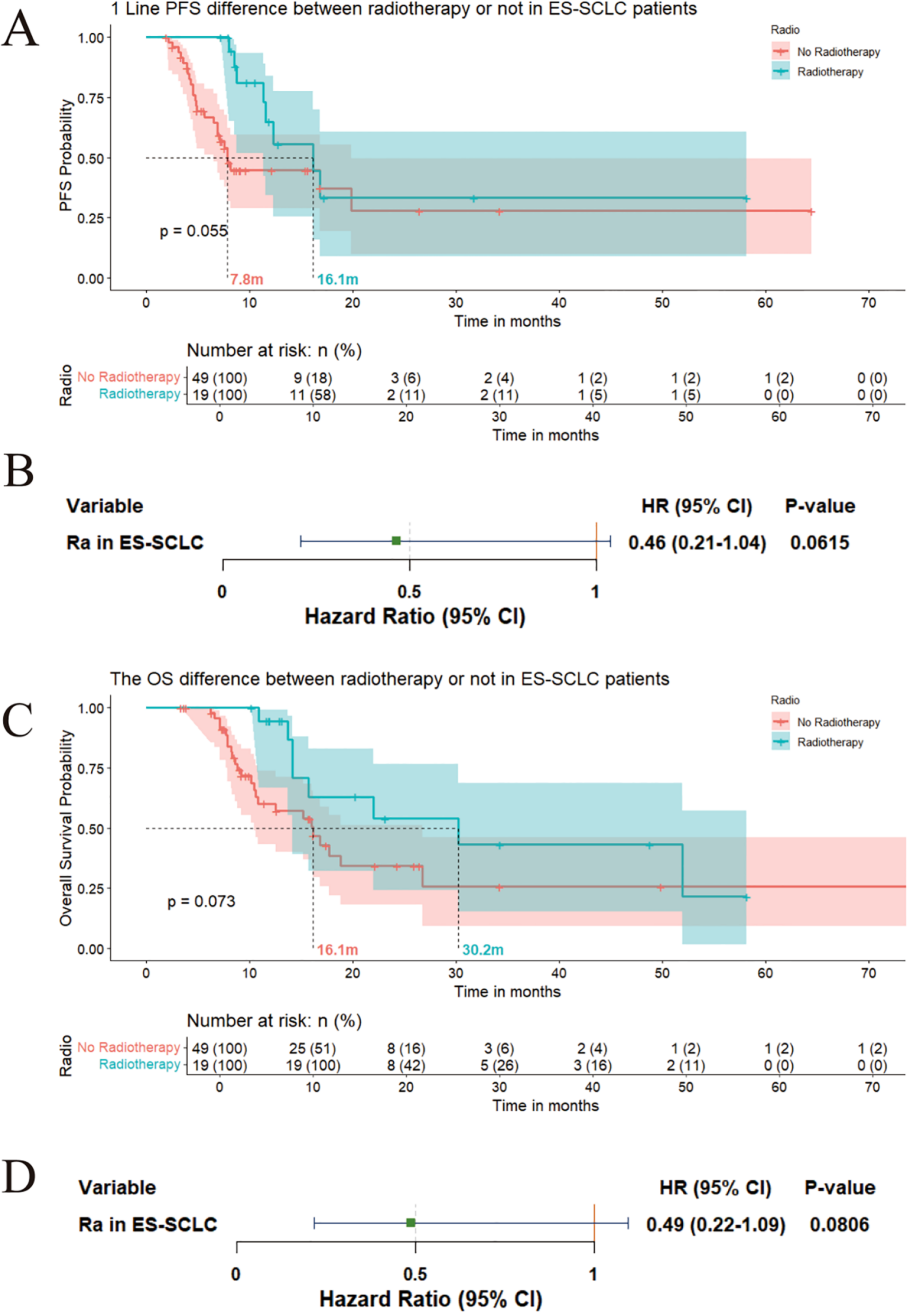
**(A)** First-line PFS comparison (irradiated vs. non-irradiated); **(B)** Cox regression for PFS benefit (HR:0.46); **(C)** OS comparison (radiotherapy vs. non-radiotherapy); **(D)** Cox regression for OS benefit (HR:0.49).

Current evidence on radiotherapy benefits in ES-SCLC remains limited. In this retrospective cohort analysis, 19 ES-SCLC patients received radiotherapy primarily for symptomatic brain metastases presenting with persistent neurological manifestations (dizziness, nausea, and headaches) or refractory bone metastasis-related pain inadequately controlled by pharmacotherapy. For patients with extensive-stage small cell lung cancer (ES - SCLC), the palliative radiotherapy protocol entails a fractionated dose of 3 Gy per fraction, amounting to a total cumulative dosage of 30 Gy for bone or brain metastases, and a fractionated dose of 2 Gy per fraction, resulting in a total cumulative dosage of 50 Gy for chest tumors. Comparative analysis indicated a marginally significant prolongation of first-line PFS in irradiated patients compared to non-irradiated patients (16.1 months vs. 7.8 months; P = 0.06) (**Figure 6A**). Cox regression analysis revealed a suggestive tendency towards a PFS benefit (HR: 0.46, 95% CI: 0.21 - 1.04) (**Figure 6B**). Moreover, patients who received irradiation demonstrated a prolonged overall survival (OS) in comparison to their non - irradiated counterparts (30.2 months vs. 16.1 months) (**Figure 6C**). This finding was corroborated by Cox analysis, which indicated a similar trend of OS advantage (HR: 0.49, 95% CI: 0.22 - 1.09) (**Figure 6D**).

## 4 Discussion

Small cell lung cancer (SCLC) remains a challenging malignancy with limited therapeutic breakthroughs, though recent advancements have modestly improved outcomes (7). Current first-line therapy for both limited-stage (LS) and extensive-stage (ES) disease relies on platinum-etoposide combinations, with the integration of immune checkpoint inhibitors in ES-SCLC demonstrating survival benefits in phase III trials (5). For LS-SCLC, concurrent chemoradiotherapy (CCRT) remains the standard, supported by meta-analyses showing a 5–10% absolute survival advantage over sequential approaches (12). Prophylactic cranial irradiation (PCI) continues to reduce brain metastasis rates, though its survival impact in ES-SCLC remains debated amid advances in MRI surveillance (13). Second-line options, such as topotecan or irinotecan, provide limited efficacy, while third-line anlotinib—a multi-targeted tyrosine kinase inhibitor—has emerged as a guideline-recommended option with modest PFS gains (9–11, 14). Despite these strides, rapid relapse, cumulative toxicities, and a paucity of targeted therapies underscore unmet needs. Our study’s findings align with and extend these observations, particularly in real-world survival outcomes and the evolving role of radiotherapy across disease stages.

The findings of this retrospective analysis provide critical insights into the differential outcomes of second-line therapy in SCLC based on platinum sensitivity, while reinforcing the prognostic value of staging and the survival benefits conferred by consolidative radiotherapy strategies. Our results underscore the importance of risk stratification and personalized therapeutic approaches in this aggressive malignancy, particularly in the context of limited treatment options beyond first-line therapy.

The study highlights a stark divergence in outcomes between platinum-sensitive (PS, 180≥TFI ≥90 days) and platinum-resistant (PR, TFI <90 days) patients. PS patients rechallenged with platinum-based regimens exhibited significantly prolonged median OS compared to non-platinum regimens (17.7 vs. 12.5 months, *p* < 0.05), aligning with ESMO guidelines (11) advocating platinum reintroduction in this subgroup. This benefit likely stems from retained tumor susceptibility to platinum-induced DNA damage and synergistic effects with salvage chemotherapy. In contrast, PR patients derived no survival advantage from platinum rechallenge, reflecting intrinsic or acquired resistance mechanisms such as enhanced DNA repair or reduced drug accumulation (4). Notably, the irinotecan-lobaplatin (IL) combination failed to improve outcomes in either subgroup, with a concerning trend toward reduced survival. These observations suggest that merely recycling cytotoxic agents—particularly those sharing resistance pathways with prior therapies—may exacerbate toxicity without overcoming resistance (15, 16). The IL regimen’s inefficacy, despite theoretical synergy between topoisomerase inhibitors and platinum, may reflect overlapping toxicities limiting dose intensity.

The analysis reaffirms the pivotal role of CCRT and PCI in optimizing outcomes across disease stages.In the context of limited-stage small-cell lung cancer (LS - SCLC), concurrent chemoradiotherapy (CCRT) exhibited a clinically significant overall survival (OS) advantage compared to sequential therapy (54.9 months versus 50.2 months;HR = 0.72, *p* = 0.08). This finding is in line with meta - analyses, which indicate that concurrent treatment approaches yield an absolute survival gain of 5 - 10% (11). This benefit likely arises from radiotherapy’s dual mechanisms: direct cytoreduction of chemoresistant clones and immunogenic cell death enhancing systemic T-cell responses. Likewise, PCI could significantly decrease the risk of brain metastasis and was correlated with an extended OS (50.2 months vs. 36.4 months, HR = 0.47), which validates its selective efficacy in patients attaining complete remission. While recent debates question PCI’s universal application—particularly given advanced MRI surveillance—our data support its integration into risk-adapted algorithms (13). The NCCN-endorsed strategy of reserving PCI for MRI-negative LS-SCLC patients with robust chemotherapy responses balances survival benefits against neurocognitive risks, a approach mirrored in ongoing trials like MAVERICK (NCT04155034) (17).

The stark contrast in PS vs. PR outcomes underscores the need for dynamic biomarkers beyond TFI to guide second-line therapy. While TFI remains a pragmatic clinical tool, emerging biomarkers such as circulating tumor DNA (ctDNA) mutational burden (18) or SLFN11 expression could refine sensitivity prediction (19). The null effect of IL regimens across subgroups further highlights the futility of empiric cytotoxic combinations without mechanistic rationale. Instead, the striking efficacy of novel agents like DLL3-targeted BiTE therapy (tarlatamab, ORR 40%) (20) and PARP inhibitors in SLFN11-high tumors—as reported in CheckMate 032 (21, 22).

Immunotherapy has demonstrated significant efficacy in small cell lung cancer (SCLC), including both LS-SCLC and ES-SCLC. Particularly in LS-SCLC, consolidation treatment with durvalumab has notably improved overall survival and progression-free survival (23, 24). In extensive-stage SCLC, combination therapy with immunotherapy and chemotherapy has also significantly enhanced patient prognosis, although the overall survival remains limited (7).Radiotherapy’s systemic immunomodulatory effects warrant particular emphasis. The prolonged PFS observed in ES-SCLC patients receiving symptom-directed radiotherapy (16.1 vs. 7.8 months) aligns with preclinical evidence of radiation-induced PD-L1 upregulation and myeloid-derived suppressor cell depletion (25). These findings suggest that localized radiotherapy may potentiate immunotherapy responses through abscopal effects. Future studies should prioritize sequencing strategies to maximize immune activation while mitigating overlapping toxicities.

This study’s retrospective design and modest sample size (n=132) limit statistical power to detect subtle survival differences. Heterogeneity in radiotherapy protocols and inconsistent biomarker profiling further constrain mechanistic interpretations.

There also exist certain analyses with p - values marginally exceeding 0.05, along with inevitable treatment bias. However, upon comprehensive consideration of all analyses, it is still posited that this conclusion is robust. In summary, this analysis validates platinum sensitivity as a crucial determinant of second - line therapy outcomes, while casting doubt on the efficacy of non - targeted cytotoxic combinations such as IL regimens. Concurrent chemoradiotherapy (CCRT) and prophylactic cranial irradiation (PCI) remain fundamental interventions for limited - stage small cell lung cancer (LS - SCLC), and emerging evidence supports the immunomodulatory role of radiotherapy in extensive - stage (ES) disease. Given the retrospective nature of the study, cautious interpretation of the findings is necessary. Nevertheless, the results offer a framework for optimizing therapeutic stratification and integrating novel agents. As the management of small cell lung cancer (SCLC) enters an era of biomarker - driven therapy, prospective validation of these insights is imperative to translate incremental survival improvements into meaningful patient outcomes.

## Data Availability

Publicly available datasets were analyzed in this study. This data can be found here: The data could be provided for reasonable requirement.
