# Supplementary figures and images for: Real-world survival patterns and multimodal therapy utilization in small cell lung cancer: a retrospective cohort study in a Chinese countryside hospital
Source: Front Oncol. 2025 Oct 23;15:1636533. doi: 10.3389/fonc.2025.1636533 (PMC12588810; doi:10.3389/fonc.2025.1636533)

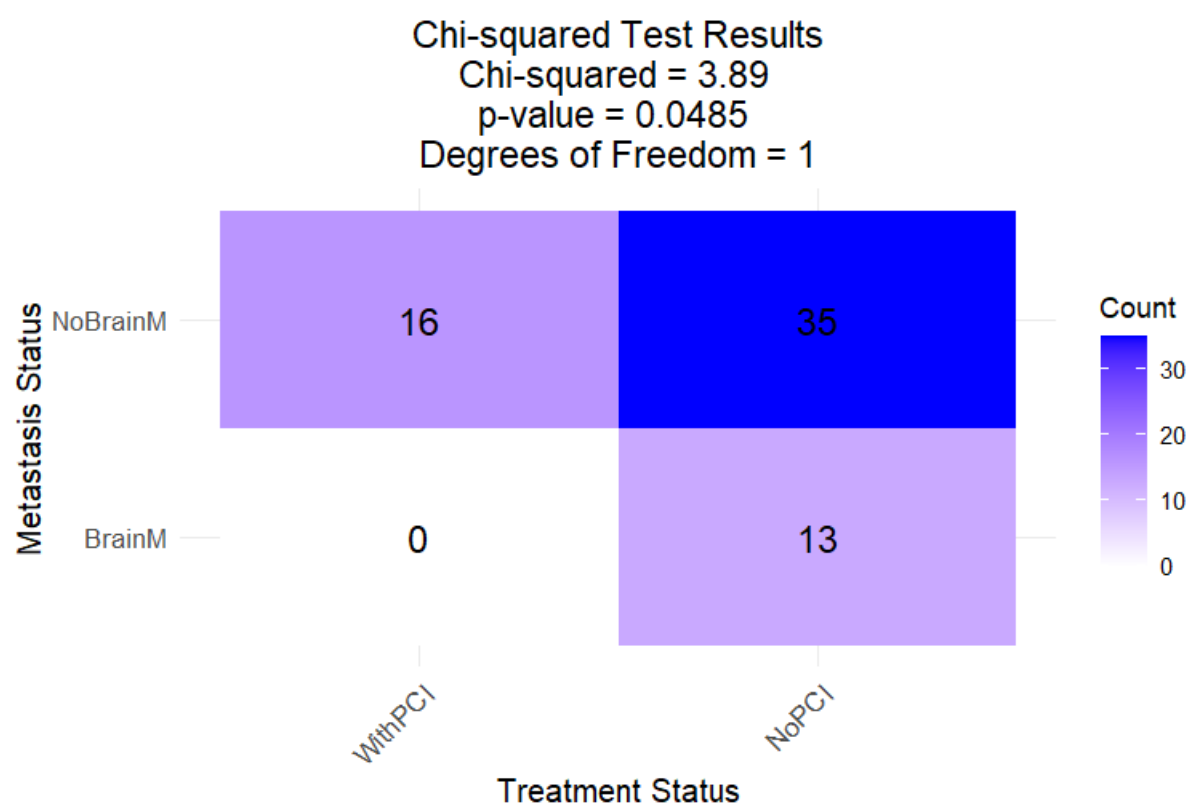

Supplement: Supplementary file 1 [file DataSheet1.pdf]

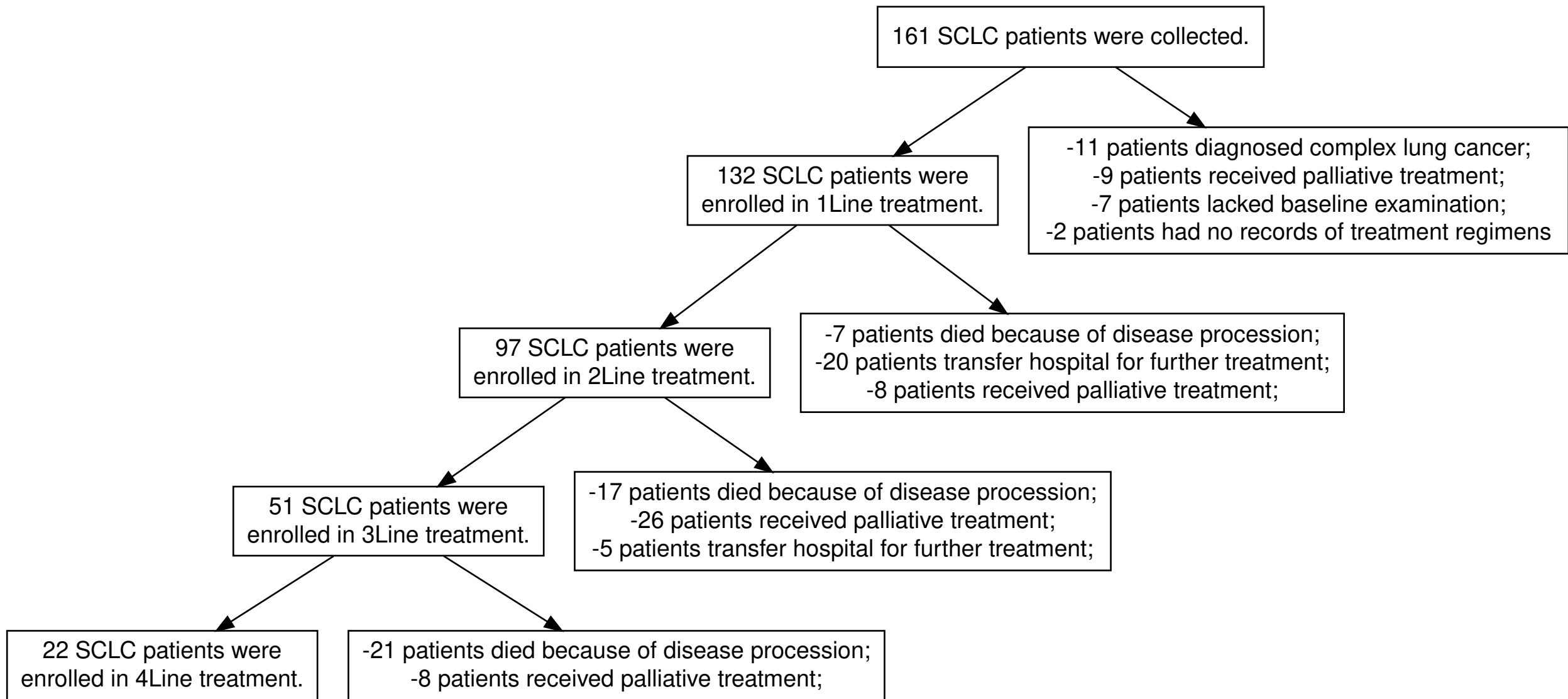

Supplement: Supplementary file 2 [file Image1.pdf]
